# Biomarker Detection and Validation for Corneal Involvement in Patients With Acute Infectious Conjunctivitis

**DOI:** 10.1001/jamaophthalmol.2024.2891

**Published:** 2024-08-15

**Authors:** Gerami D. Seitzman, Lalitha Prajna, N. Venkatesh Prajna, Wiwan Sansanayudh, Vannarut Satitpitakul, Wipada Laovirojjanakul, Cindi Chen, Lina Zhong, Kevin Ouimette, Travis Redd, Michael C. Deiner, Travis C. Porco, Stephen D. McLeod, Thomas M. Lietman, Armin Hinterwirth, Thuy Doan

**Affiliations:** 1 Francis I Proctor Foundation, University of California, San Francisco; 2 Department of Ophthalmology, University of California, San Francisco; 3 Department of Ocular Microbiology, Aravind Eye Hospital, Madurai, Tamil Nadu, India; 4 Phramongkutklao Hospital, Bangkok, Thailand; 5 Center of Excellence for Cornea and Stem Cell Transplantation, Department of Ophthalmology, Faculty of Medicine, Chulalongkorn University, Bangkok, Thailand; 6 Thai Red Cross Society, Bangkok, Thailand; 7 Khon Kaen University, Khon Kaen, Thailand; 8 Oregon Health & Science University, Portland; 9 Department of Epidemiology and Biostatistics, University of California, San Francisco; 10 Institute for Global Health Sciences, University of California, San Francisco

## Abstract

**Importance:**

Infectious conjunctivitis can lead to corneal involvement and result in ocular morbidity. The identification of biomarkers associated with corneal involvement has the potential to improve patient care.

**Objective:**

To identify biomarkers in patients with acute infectious conjunctivitis.

**Design, Setting, and Participants:**

This cross-sectional study took place from December 2016 to March 2024. Analyses were performed in 3 phases. First, logistic regression and machine learning algorithms were used to predict the probability of demonstrating corneal involvement in patients with presumed infectious conjunctivitis. Second, quantitative reverse transcription polymerase chain reaction (RT-qPCR) was used to confirm the most important biomarker gene identified by the algorithm. Third, the biomarker gene was validated in prospectively collected conjunctival samples of adult patients from 3 outpatient centers in Thailand and 1 in India. Patients with signs and symptoms of infectious conjunctivitis and onset within less than 14 days were eligible. Exclusion criteria were the inability to consent, presumed toxicity, or allergic conjunctivitis.

**Exposures:**

Acute infectious conjunctivitis.

**Main Outcomes and Measures:**

The identification and validation of ocular surface gene expression associated with corneal findings on slitlamp examination.

**Results:**

Thirteen genes exhibited a 1.5–log_2_ fold change in expression in patients with corneal involvement compared to patients without corneal involvement. Using the 13 genes to train and cross validate, logistic regression produced the highest mean area under the receiver operating characteristic curve (AUROC; 0.85; 95% CI, 0.84-0.86) for corneal involvement. The removal of apolipoprotein E (*APOE*) from the gene ensemble led to a decline in predictive performance of the logistic regression classifier (from mean AUROC 0.85 [95% CI, 0.84-0.86] to 0.74 [95% CI, 0.73-0.75]; adjusted *P* = .001 [Tukey test]). Orthogonal testing of *APOE* expression level with RT-qPCR showed that *APOE* expression was higher in patients with corneal involvement compared to patients without (median [IQR], 0.23 [0.04-0.47] vs 0.04 [0.02-0.06]; *P* = .004 [Mann-Whitney *U* test]). Using a Youden index of 0.23 Δ threshold cycle, *APOE* had a sensitivity of 56% (95% CI, 33-77) and a specificity of 88% (95% CI, 79-93) in 106 samples with conjunctivitis at Aravind, India (*P* < .001 [Fisher exact test]). When applied to a different patient population in Thailand, the same criteria could discriminate between disease states (58 samples; sensitivity, 47%; 95% CI, 30-64 and specificity, 93%; 95% CI, 77-99; *P* = .001 [Fisher exact test]).

**Conclusions and Relevance:**

The results from this study suggest that the host conjunctival immune response can be meaningfully interrogated to identify biomarkers for ocular surface diseases.

## Key Points

**Question:**

Can biomarkers that may be important to clinical outcomes in patients with infectious conjunctivitis be identified?

**Findings:**

In this cross-sectional study that leveraged a combination of high-throughput sequencing, predictive algorithms, and various orthogonal validation approaches, apolipoprotein E (*APOE*) was identified as a biomarker associated with corneal involvement. *APOE* was highly discriminant between disease states in patients tested in India and Thailand.

**Meaning:**

These findings suggest that relevant and minimally invasive ocular biomarkers can be identified to facilitate precision care.

## Teaser

This cross-sectional study evaluates potential biomarkers in patients with acute infectious conjunctivitis.

## Introduction

Infectious conjunctivitis can lead to vision loss when the cornea becomes involved and inflamed and conjunctivitis outbreaks remain a major public health burden.^1^ The rate of corneal involvement with infectious conjunctivitis is variable, can affect any sociodemographic group, and may be pathogen and serotype dependent.^2^

Signs of corneal involvement with conjunctivitis can range from mild (ie, punctate epithelial erosions or keratopathy) to more severe, such as subepithelial corneal infiltrates (SEIs). Punctate epithelial erosions represent corneal epithelial cell loss while SEIs represent an overactive postinfectious inflammatory process that presents clinically as patchy opacification in the anterior corneal stroma just beneath the corneal epithelial layer. SEIs are the hallmark corneal manifestation of epidemic keratoconjunctivitis outbreaks. Here, the presumptive causative pathogens are viruses, commonly adenoviruses, and postviral SEIs can be chronic and recurrent.^3^ Postinfectious, visually significant SEIs may occur with other pathogens, including microsporidia (commonly preceded by raised epithelial lesions),^4,5^ herpes viridae,^6,7^ and SARS-CoV-2.^8,9^ Although some pathogens make SEI development more likely, there is presently no way to predict their occurrence. Therapeutic interventions for corneal involvement include the use of topical immunosuppressives, which may clear the opacities.^3,10^ Patients with corneal involvement may progress to vision loss. It is still unclear if preventive treatment can halt or minimize the occurrence and reoccurrence of corneal sequelae.

The term *biomarker* refers to an objective measure that can be used to predict a condition or disease. An effective biomarker allows for the prediction of both occurrence and severity of a disease and, ideally, identifies a population where treatment can be initiated to minimize the negative impact of a disease.^11^ Because infectious conjunctivitis causes an intense inflammatory response in a relatively accessible organ, the disease provides an ideal opportunity to characterize the local host immune response. Prior work^12^ has shown that we could identify transcriptome signatures associated with pathogen types. In addition, the output of metagenomic datasets is large, thus making machine learning potentially optimal for biomarker identification and prediction within these data repositories.^13,14,15^ The objective of this study was to use RNA-seq, various statistical classifier models, and directed quantitative reverse transcription polymerase chain reaction (RT-qPCR) to develop a model that can identify conjunctival biomarkers associated with corneal involvement in patients with acute infectious conjunctivitis.

## Methods

### Patient Selection

Conjunctival swabs were obtained from patients with presumed infectious conjunctivitis who presented to the Proctor Foundation at the University of California, San Francisco (UCSF); the Aravind Eye Hospital in Madurai, India; Phramongkutklao Hospital, Bangkok, Thailand; Chulalongkorn University, Bangkok, Thailand; and Khon Kaen University, Khon Kaen, Thailand. Inclusion criteria included having symptoms suggestive of infectious conjunctivitis for less than 14 days.^12^ Patients were excluded if suspected to have allergic or toxic conjunctivitis. Sex and age were self-reported. This study adhered to the tenets of the Declaration of Helsinki. The institutional review board of UCSF and the ethics review committees of Aravind Eye Hospital, Phramongkutklao Hospital, Chulalongkorn University, and Khon Kaen University approved the study. Informed written consent was obtained from all patients and no incentives were given.

### Sample Collection

The lower fornix of the affected eye was swept 2 times using a sterile polyester-tipped applicator (Puritan). The swab was placed in DNA/RNA-Shield (Zymo Research) to preserve the integrity of the nucleic acids in the sample. Samples from India and Thailand were stored at −20 °C until shipment to UCSF for long-term storage at −80 °C.

### Patient Populations

Fifty-eight convenience samples from 54 presumed infectious conjunctivitis patients from Madurai, India, and San Francisco, California, collected between December 2016 and January 2020, were used for phases 1 and 2 (eTable 1 in Supplement 1). In phase 3, prospectively collected samples from patients enrolled in the Seasonal Conjunctivitis Outbreak Reporting for Prevention and Improved Outcomes (SCORPIO) study at Aravind Eye Hospital, Phramongkutklao Hospital, Chulalongkorn University, and Khon Kaen University from April 2021 to December 2022 were included for analysis (eTable 3 in Supplement 1).

### Laboratory Methods

Samples were deidentified and randomized prior to sample processing. Researchers processing and analyzing the samples were masked to deidentify patient information. Nucleic acid extraction and sequencing libraries were prepared and sequenced as previously described.^12,16^ In brief, total RNA was extracted from the conjunctival samples using the Quick-DNA/RNA Microprep Plus Kit (Zymo Research) per the manufacturer’s instructions. From each sample, 5 μL of extracted total nucleic acids were first converted to complementary DNA (cDNA), and sequencing libraries were prepared using the NEBNext RNA ULTRA IIp Kit (New England Biolabs) according to the manufacturer’s instructions and then amplified with 16 PCR cycles. Samples were sequenced on the NovaSeq system (NovaSeq 6000; Illumina) using 150 nucleotide–paired end sequencing.

### Identification of Corneal Involvement

Patients seen at the outpatient clinic at the participating sites were examined using slitlamp biomicroscopy by trained ophthalmologists at presentation. For this study, the definition of corneal involvement is broad and includes abnormal corneal surface staining with fluorescein, raised corneal epithelial lesions, and SEIs.

### Data Analysis

Differential host gene expression analysis was performed as previously described.^17,18^ Briefly, sequencing reads were quality filtered and aligned to the GRCh38 human genome assembly using HISAT2 version 2.1.0. Abundance of transcripts was calculated using the default parameters in stringtie2 version 1.3.4d and annotation of transcripts was based on ENSEMBL GRCh38.87. Gene count matrices were generated using stringtie2’s prepDE.py script according to the protocol found in the stringtie2 documentation (Python 3.6.7). Gene count data were analyzed in a set of 46 samples (eTable 1 in Supplement 1) with DESeq2 version 1.28.1 to evaluate for differences between patients with corneal involvement and those without corneal involvement. Genes for which DESeq2 reported an adjusted *P* value (false discovery rate) less than .05 and with at least a log_2_ fold change of 1.5 were considered notable.

### RT-qPCR

Transcript expression for individual genes was quantified. RT-qPCR runs were performed in a Mic qPCR Cycler (Bio Molecular Systems). cDNA was synthesized using the SuperScript VILO cDNA Synthesis Kit (Invitrogen). Five microliters of RNA were added into a first-strand cDNA synthesis reaction per the manufacturer’s recommendations. Two microliters of cDNA were added to each PCR reaction mix (20 μL), containing 10 μL of 2X TaqMan Fast Advanced Master Mix (Applied Biosystems), 1 μL of ApoE 20X TaqMan Gene Expression Assay (Hs00171168_m1, Thermo Fisher) or 1 μL GAPDH 20X TaqMan Gene Expression Assay (Hs02786624_g1, Thermo Fisher), and 7 μL of nuclease-free water. The following protocol was used: an initial uracil-N-glycosylase incubation step at 50 °C for 2 minutes and a polymerase activation/cDNA denaturation step at 95 °C for 2 minutes, followed by 40 cycles of 95 °C for 3 seconds and 60 °C for 30 seconds. All samples were processed in duplicates. Data were acquired using micPCR software version 2.6.5 (Bio Molecular Systems). *APOE* expression values were normalized to glyceraldehyde 3-phosphate dehydrogenase (GAPDH) levels using the following equation: 2^(CT[GAPDH] − CT[^*^APOE^*^])^.

### Statistical Analyses

DESeq2-normalized counts of the differentially expressed genes of an expanded sample set (n = 58; eTable 1 in Supplement 1) were used to train and validate various classifier models (scikit-learn, Python library, version 1.4.1). Since the number of samples was small, we used a randomized subsampling approach to get a measure of the trainability of each model. In each of 1000 iterations, the set of samples was split 70/30 using the scikit-learn function *StratifiedShuffleSplit*, which preserves the percentage of samples for each class. Features were standardized using a default *StandardScaler* function, which removes the mean and scales to unit variance. Logistic regression, random forest, linear support vector machine, and decision tree models with the default parameters defined in scikit-learn were then trained on the training fold and validated on the validation fold. We used the mean area under the receiver operating characteristic curves (AUROCs) to estimate the reliability of the performance metrics and compared the AUROCs using the method by Hanley and McNeil.^19^

To identify genes predictive of corneal involvement, we compared the predictive performance between a full model and one where that gene was held out.^20^ To assess the statistical significance of differences in the distributions of AUROC values across various scenarios, an analysis of variance test was conducted. *P* values for the multiple pairwise comparisons were calculated using Tukey procedure (statsmodel, Python library, version 0.14.1). In addition, SHAP (Shapley Additive Explanations) values were calculated for each classifier model to rank the influence of each gene on the model output (SHAP library v0.45.1).

The fold changes of the apolipoprotein E (*APOE*) RT-qPCR results of phase 2 were fitted to a logistic regression model, and the Youden *J* statistic was used to determine the best fold change threshold to distinguish the 2 classes. For phase 3, the *APOE* RT-qPCR was performed on extracted RNA from conjunctival samples of patients who participated in the SCORPIO Study Group. Sensitivity and specificity were estimated using a 2 × 2 table (confusion matrix). The Fisher exact test was used to determine the discriminability of *APOE* RT-qPCR with clinical findings of corneal involvement (GraphPad version 10). This study adhered to the Transparent Reporting of a Multivariable Prediction Model for Individual Prognosis or Diagnosis (TRIPOD) reporting guideline.

## Results

**Figure 1.**
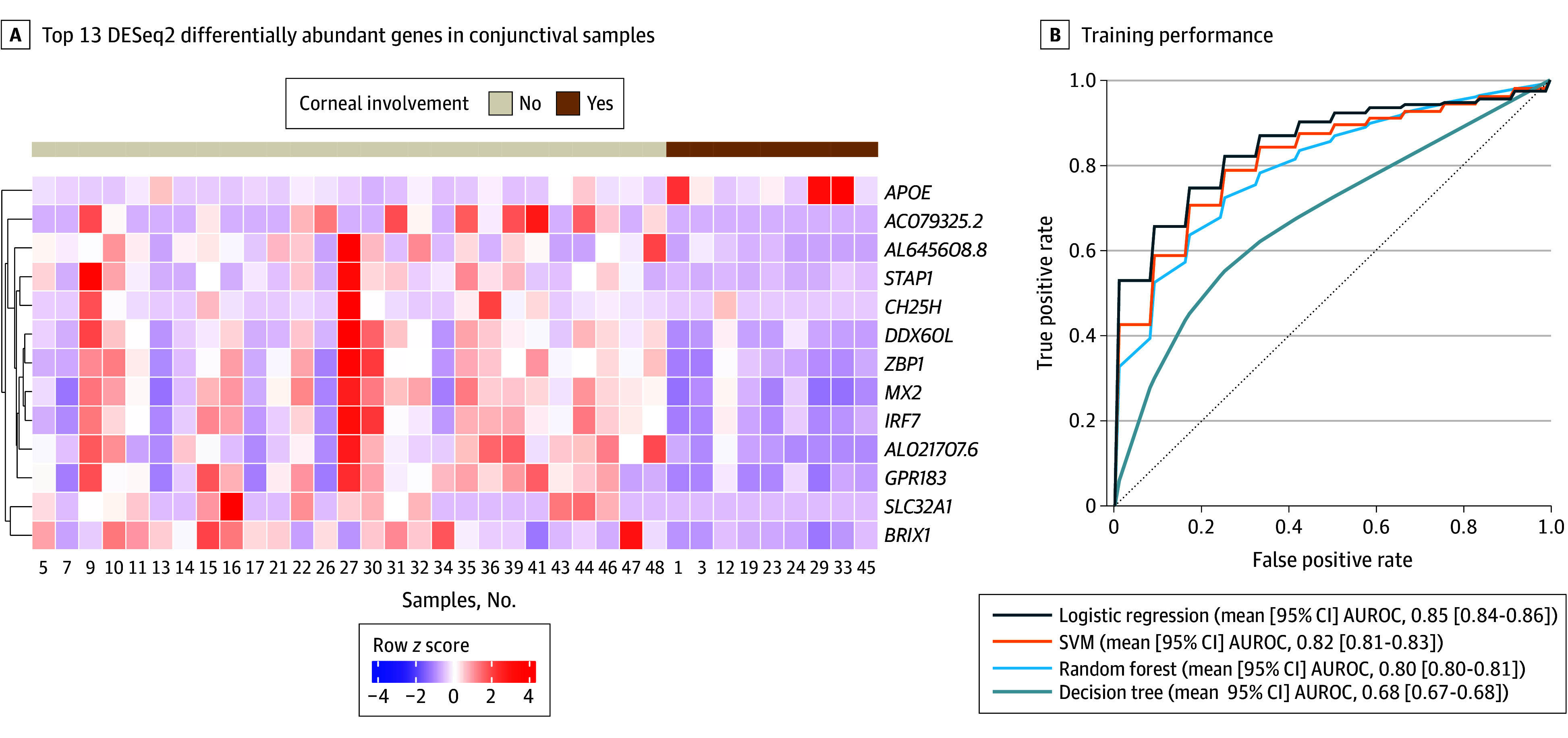
Phase 1 of the Analytic Protocol A, Heatmap showing the top 13 DESeq2 differentially abundant genes in conjunctival samples from patients with presumed infectious conjunctivitis with and without corneal involvement. B, Training performance of logistic regression, random forest, decision tree, and linear support vector machine (SVM). AUROC indicates area under the receiver operating characteristic curve.

**Figure 2.**
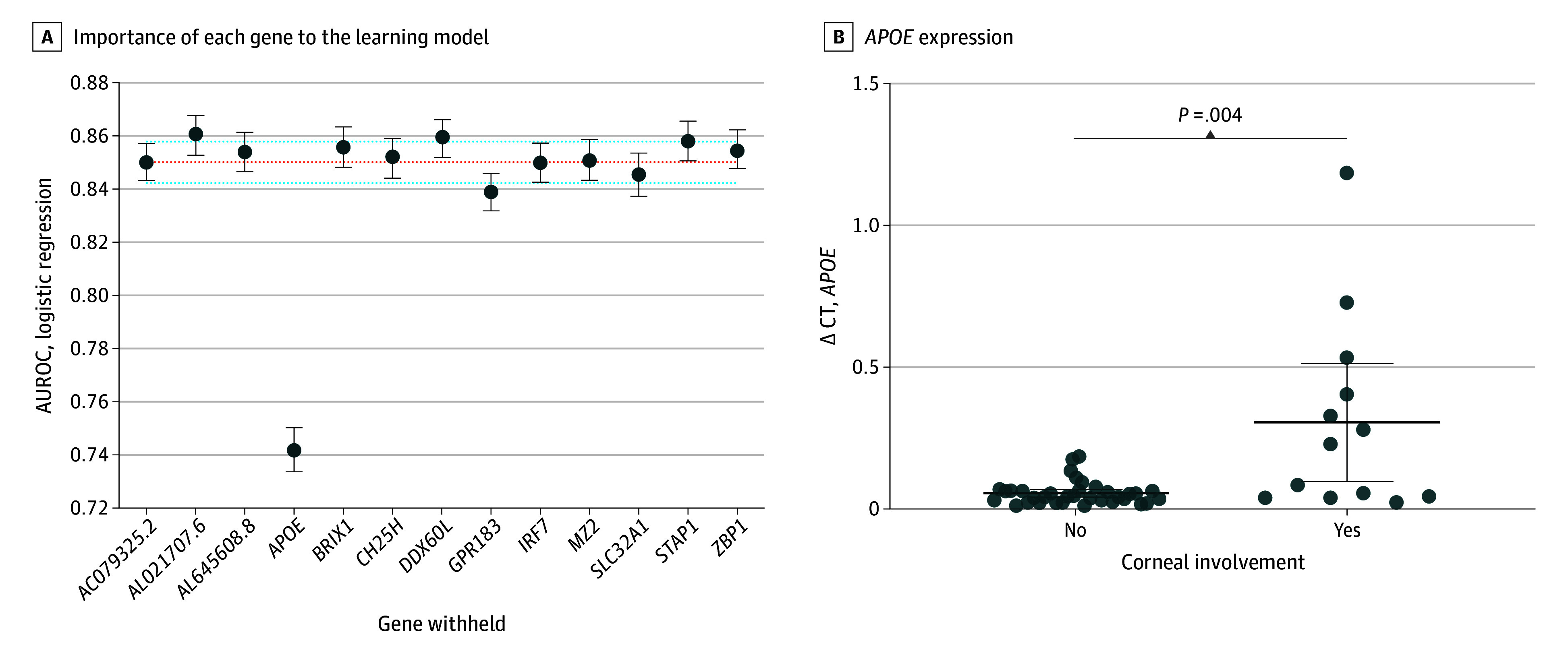
Phase 2 of the Analytic Protocol A, Each data point represents the mean area under the receiver operating characteristic curve (AUROC) of logistic regression models that left out the respective gene over 1000 randomized training and validation splits. The red dotted line represents the mean AUROC for all splits of the full model of all 13 genes. The blue dotted lines represent the 95% CIs of the full model. Error bars represent 95% CIs for the individual models. B, Validation of *APOE* in conjunctival samples of 48 patients with presumed acute infectious conjunctivitis using quantitative reverse transcription polymerase chain reaction (RT-qPCR) analysis. *APOE* expression was normalized to glyceraldehyde 3-phosphate dehydrogenase and expressed as Δ threshold cycle (Δ CT). *P* value was calculated using the Mann-Whitney *U* test.

This study was conducted in 3 phases (eFigure 1 in Supplement 1). The first phase was to identify a set of genes associated with patients who demonstrate corneal involvement with slitlamp biomicroscopy. To do this, we performed differential analysis on human transcripts using RNA-seq from conjunctival samples of 36 patients with presumed infectious conjunctivitis who presented to the Aravind Eye Hospital (eTable 1 in Supplement 1). Thirteen genes were found to be differentially expressed between patients with corneal involvement (n = 9) compared to those without (n = 27) (Figure 1A; eTables 1 and 2 in Supplement 1). We then used models to predict the probability of corneal involvement using the gene signatures. Models were trained and cross validated on data from 58 samples collected from 54 patients (20 samples with corneal involvement and 38 samples without corneal involvement) (eTable 1 and eFigure 2 in Supplement 1). Of the 4 models used, standard logistic regression produced the highest mean (95% CI) AUROC (0.85 [0.84-0.86]) compared to the support vector machine (0.82 [0.81-0.83]), random forest (0.80 [0.80-0.81]), and decision tree (0.68 [0.67-0.68]) models for corneal involvement (Figure 1B). The logistic regression classifier was chosen for further analysis. The model’s performance dropped after *APOE* was removed (mean AUROC, 0.85 [95% CI, 0.84-0.86] vs 0.74 [95% CI, 0.73-0.75]; adjusted *P* = .001 [Tukey test]), indicating *APOE* may be an important biomarker associated with corneal involvement (Figure 2). Similarly, *APOE* appeared to have the highest impact SHAP values (eFigure 3 in Supplement 1).

In the second phase, we orthogonally validated that the expression of *APOE* was correlated with clinical findings in a subset of conjunctival samples used in training and validation. RT-qPCR was performed on 48 samples (13 with and 35 without corneal involvement) (eTable 1 in Supplement 1). The normalized expression of *APOE* was higher in the samples from patients with corneal involvement compared to samples from patients without corneal involvement (median [IQR], 0.23 [0.04-0.47] vs 0.04 [0.02-0.06]; *P* = .004 [Mann-Whitney *U *test]) (Figure 2B). From the same dataset, we generated a ROC curve to determine the optimal Δ threshold cycle for corneal involvement. The Youden index was a 0.23-fold change in *APOE* expression.

**Figure 3.**
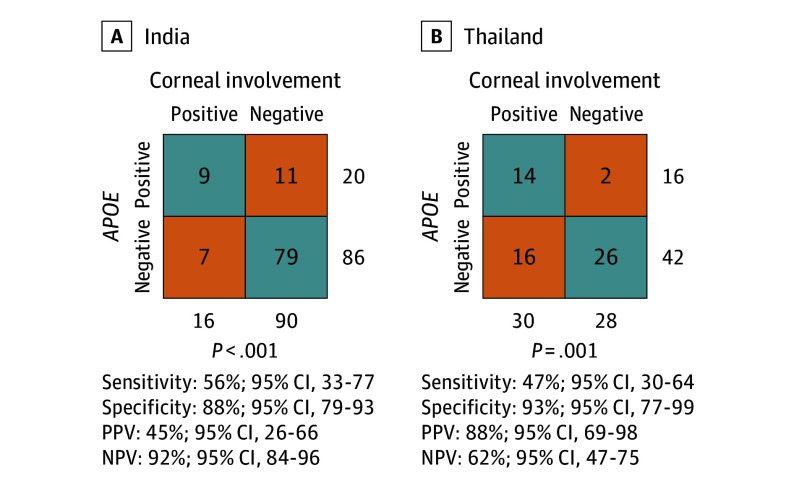
Phase 3 of the Analytic Protocol Performance of *APOE* as a marker for corneal involvement in the conjunctival samples of patients with presumed acute infectious conjunctivitis in India and Thailand. *P* values were calculated using the Fisher exact test. NPV indicates negative predictive value; PPV, positive predictive value.

In the third phase, we validated the findings in patients whose samples were collected prospectively (eTable 3 in Supplement 1). These were patients who participated in the SCORPIO Study and their samples were not used for training or validation. SCORPIO is an international consortium to track pathogens for presumed acute infectious conjunctivitis. Sample collection and enrollment criteria for SCORPIO were the same as the prior patient cohort used in phases 1 and 2 of this analysis. We performed *APOE* RT-qPCR in patients enrolled in Aravind Eye Hospital in India and Phramongkutklao Hospital, Chulalongkorn University, and Khon Kaen University in Thailand (Figure 3). Using the threshold identified above, the sensitivity and specificity of *APOE* to classify corneal involvement for 106 patients in India was 56% (95% CI, 33-77) and 88% (95% CI, 79-93), respectively (*P* < .001 [Fisher exact test]). Similar sensitivity and specificity were observed in the patient cohort (n = 58) from 3 study sites in Thailand (47% [95% CI, 30-64] and 93% [95% CI, 77-99]; *P* = .001[Fisher exact test]).

## Discussion

In this cross-sectional study, we showed that we could identify a biomarker with high specificity for corneal involvement in patients with presumed infectious conjunctivitis by leveraging bulk RNA-seq, simple machine-learning approaches, and direct testing on multiple patient populations. Here, we found the expression level of *APOE* in the conjunctiva is associated with corneal involvement seen on examination.

*APOE* is a protein involved with the transport of lipids and cholesterol. *APOE* is the main cholesterol carrier in the brain, and the *APOE4* variant a risk factor for the development of Alzheimer disease in some populations.^21^ There is a suggestion that *APOE4* may worsen the inflammation caused by amyloid deposition.^22^ The role *APOE* plays in the cornea has not been well characterized. *APOE* may be associated with corneal dystrophies that involve visually significant lipid deposition into the cornea, such as Schnyder corneal dystrophy,^23^ as well as corneal dystrophies involving amyloid deposition.^24^ While *APOE* is associated with an increased risk of systemic herpes infections^25^ it is unclear if this risk extends to herpetic keratitis and its sequelae, although there is a suggestion of increased *APOE2* variant in this population.^26,27^ When used topically in an animal model, *APOE* peptide mimetic appeared to exhibit some antiviral properties.^28^

SCORPIO is an ongoing international consortium that tracks pathogens causing conjunctivitis worldwide. This study revealed that the pathogens causing infectious conjunctivitis are diverse and largely dependent on geography. In southern India, the predominant organisms include the human adenovirus (HAdV) and the fungus *Vittaforma corneae*, both of which can result in corneal involvement. Indeed, for *V corneae*, a common clinical presentation is the stuck-on appearance of raised corneal epithelial lesions that stain with fluorescein. The set of samples used for dimension reduction, machine learning training, and validation was collected from patients with a pathogen identified (ie, HAdV or *Vittaforma corneae*) or without a pathogen identified (ie, negative on metagenomic sequencing). Thus, the analysis was agnostic of the causative agents and focused on the corneal involvement as an outcome. From a biomarker testing standpoint, this may serve as a strength, as the detection of sequelae of the disease is the main objective.

The prevalence of corneal involvement in infectious or epidemic conjunctivitis is not well established. It appears to be pathogen dependent and ranges from around 38% to 80%, although many patients are unlikely to have a slitlamp examination on presentation, as they usually present to urgent care or their primary care physicians instead of an ophthalmologist.^29,30^ From a clinical standpoint, the positive predictive and negative predictive values may be more inherently meaningful than the sensitivity and specificity of the test. While the sensitivity and specificity of *APOE* were similar for the patient populations in Thailand and southern India, the positive predictive value and negative predictive value are likely to be different, as these values are dependent on prevalence.

While the specificity of *APOE* for corneal involvement is high, the sensitivity reported here is modest. From a translational standpoint, given that a careful slitlamp examination can reveal corneal pathology, the utility of a biomarker associated with corneal pathology is helpful in a few settings. One is when a careful slitlamp eye examination is not available, particularly in resource-poor areas or when a patient presents to primary care or an emergency department lacking in ophthalmology services. Notably, most patients with conjunctivitis seek primary care, not subspecialty care. In these settings, subjective concerns of photophobia and decreased acuity are common in patients with conjunctivitis. One potential use of a biomarker associated with corneal findings may be to identify an at-risk population that would benefit from a timely referral for a specialized examination. Assuming the *APOE* marker can be adapted to a point-of-care test, a patient with presumed infectious conjunctivitis with a positive test result may be triaged to be seen by an optometrist or ophthalmologist earlier in their disease course. Given that most health care money is spent on follow-up care for conjunctivitis, the economic feasibility of this approach could balance the cost of a point-of-care test with the money saved on decreased follow-up visits.^31^

The association of *APOE* with an increased risk for corneal involvement also suggests future work investigating correlation with clinical severity, and ability to predict disease and affect clinical management. If *APOE* can predict the development of keratitis early in the conjunctivitis course, the potential for earlier or preventive measures can be initiated. The current treatment for corneal opacities associated with conjunctivitis is judicious anti-inflammatory treatment, but the choice of agents is controversial, and outcomes are variable.^32^ Both host and agent features are potentially important contributors to treatment outcomes, and *APOE* may play a role in host cornea response and in guiding treatment.

### Strengths and Limitations

The strength of this study is the combination of various approaches, including the mining of retrospective data, high-throughput sequencing, predictive algorithms (standard logistic regression and machine learning), verification at the bench, and then direct validation of the results on prospectively collected samples in multiple patient populations. This study also has limitations. First, we did not validate the performance of combinations of genes, such as *APOE* and *GPR183 *or* APOE* and *BRX1* or other combinations, as this was not within the scope of the study. Thus, the modest sensitivity observed with just *APOE* could potentially be improved if used in combination with 1 or more genes. Feature ordering using machine learning models is likely to improve classification performance. The modest sensitivity of *APOE* observed here limits its use as a screening biomarker, although in the right context, its high specificity could still be useful in identifying patients who may benefit from a close examination by a specialist. Our definition of corneal involvement was broad, and subtypes (eg, punctate erosions, raised keratitis, and SEI) were not specified or studied separately. There was no follow-up examination information to evaluate for disease progression. The reported sensitivity and specificity may not extrapolate to regions of the world other than Thailand and India.

## Conclusions

In summary, this proof-of-concept study suggests the feasibility of identifying biomarkers relevant to ocular surface diseases. For presumed infectious conjunctivitis, regardless of pathogen types, visible corneal involvement was associated with *APOE*, and this biomarker appeared to have high specificity in patients in India and Thailand. Similar approaches may be of relevance to the identification of other biomarkers in ophthalmology.

## Notes

Supplement 1. eFigure 1. Analytic protocol schematics. eFigure 2. Pathogens identified by RNA-seq in the samples of the patients included in Phase I and II. eFigure 3. SHAP summary plot of the 13 features contributing to the logistic regression classifier (A) and the support vector machine classifier (B). eTable 1. Characteristics of samples included in Phases I and II. eTable 2. Differential gene expression between patients with and without corneal involvement. eTable 3. Characteristics of samples included in Phase III.

Supplement 2. The SCORPIO Study Group members

Supplement 3. Data sharing statement
