# Supplementary material for: Biomarker Detection and Validation for Corneal Involvement in Patients With Acute Infectious Conjunctivitis
Source: JAMA Ophthalmol. 2024 Aug 15;142(9):865–71. doi: 10.1001/jamaophthalmol.2024.2891 (PMC11327903; doi:10.1001/jamaophthalmol.2024.2891)
Supplement: Supplement 1. — eFigure 1. Analytic protocol schematics. eFigure 2. Pathogens identified by RNA-seq in the samples of the patients included in Phase I and II. eFigure 3. SHAP summary plot of the 13 features contributing to the logistic regression classifier (A) and the support vector machine classifier (B). eTable 1. Characteristics of samples included in Phases I and II. eTable 2. Differential gene expression between patients with and without corneal involvement. eTable 3. Characteristics of samples included in Phase III. [file jamaophthalmol-e242891-s001.pdf]

Supplemental Online Content

Seitzman GD, Prajna L, Prajna NV, et al; SCORPIO Study Group. Biomarker detection and validation for corneal involvement in patients with acute infectious conjunctivitis. *JAMA Ophthalmol*. Published online August 15, 2024.
doi:10.1001/jamaophthalmol.2024.2891

eFigure 1. Analytic protocol schematics

eFigure 2. Pathogens identified by RNA-seq in the samples of the patients included in Phase I and II

eFigure 3. SHAP summary plot of the 13 features contributing to the logistic regression classifier (A) and the support vector machine classifier (B)

eTable 1. Characteristics of samples included in Phases I and II

eTable 2. Differential gene expression between patients with and without corneal involvement

eTable 3. Characteristics of samples included in Phase III

This supplemental material has been provided by the authors to give readers additional information about their work.

© 2024 Seitzman GD et al. *JAMA Ophthalmology*.

eFigure 1. Analytic protocol schematics. A) Phase I: dimension reduction and training of models using transcripts obtained with RNA-seq on conjunctival samples of patients with presumed infectious conjunctivitis. B) Phase II: orthogonal validation using RT-qPCR of the candidate gene and threshold selection. C) Phase III: validation of candidate gene on conjunctival samples of patients enrolled in an international conjunctivitis consortium (SCORPIO Study Group).

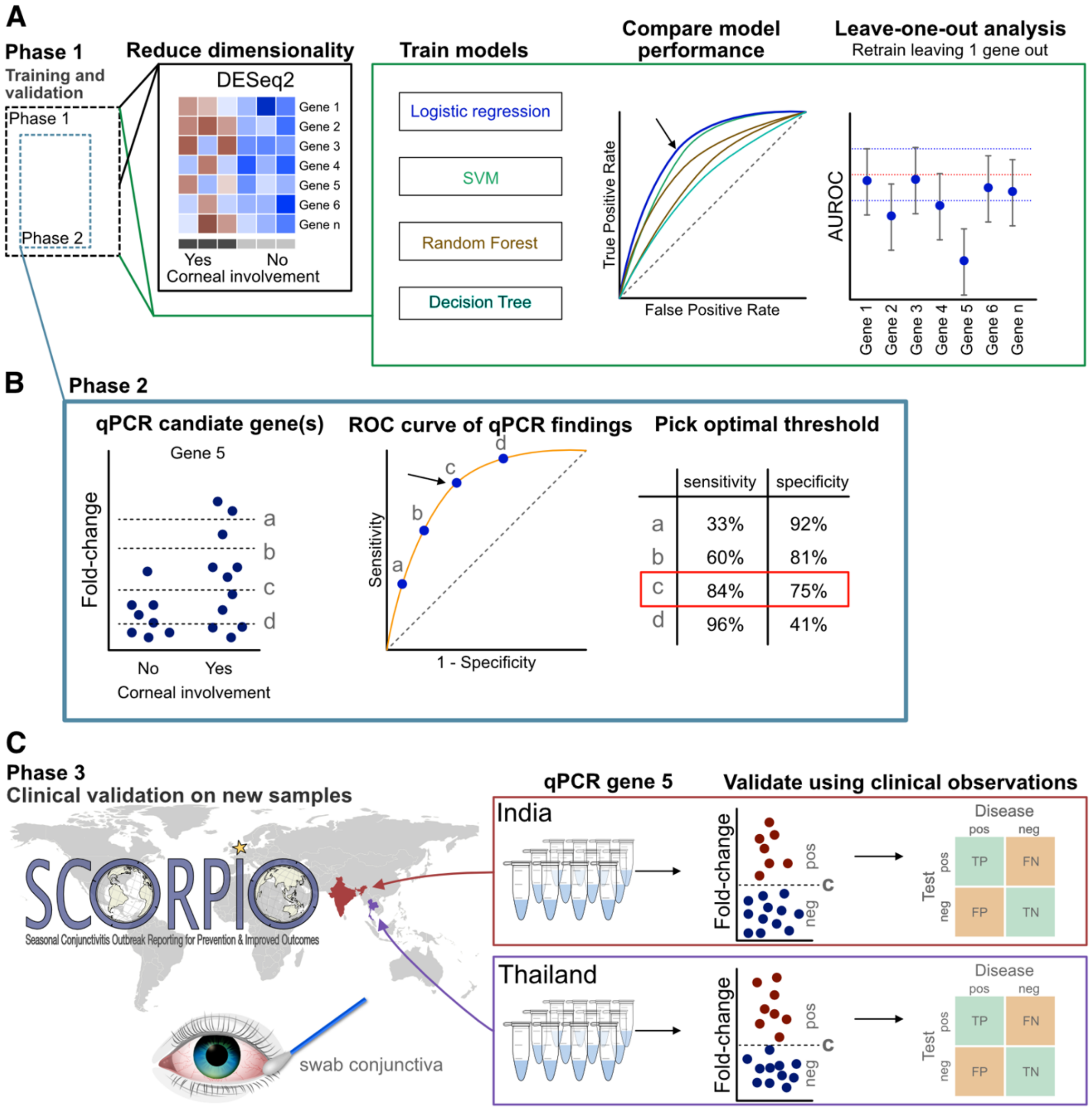

© 2024 Seitzman GD et al. *JAMA Ophthalmology*.

eFigure 2. Pathogens identified by RNA-seq in the samples of the patients included in Phase I and II.

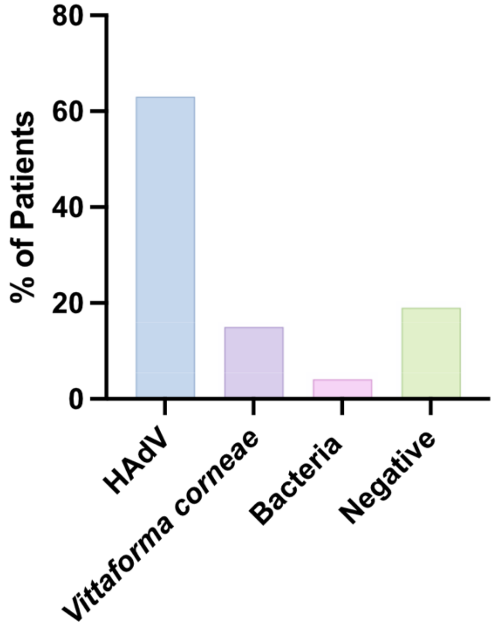

© 2024 Seitzman GD et al. *JAMA Ophthalmology*.

eFigure 3. SHAP (Shapley Additive explanation) summary plot of the 13 features contributing to the logistic regression classifier (A), the linear support vector machine classifier (B), random forest classifier (C), and decision tree classifier (D). The beeswarm plots on the left side of each panel show how features (genes) in the dataset impact a model's output. Each sample is represented by a dot, whose x-position is determined by its SHAP value. The color indicates the original value of each feature. The bar plot on the right gives a ranked overview of the maximum SHAP values for each feature.

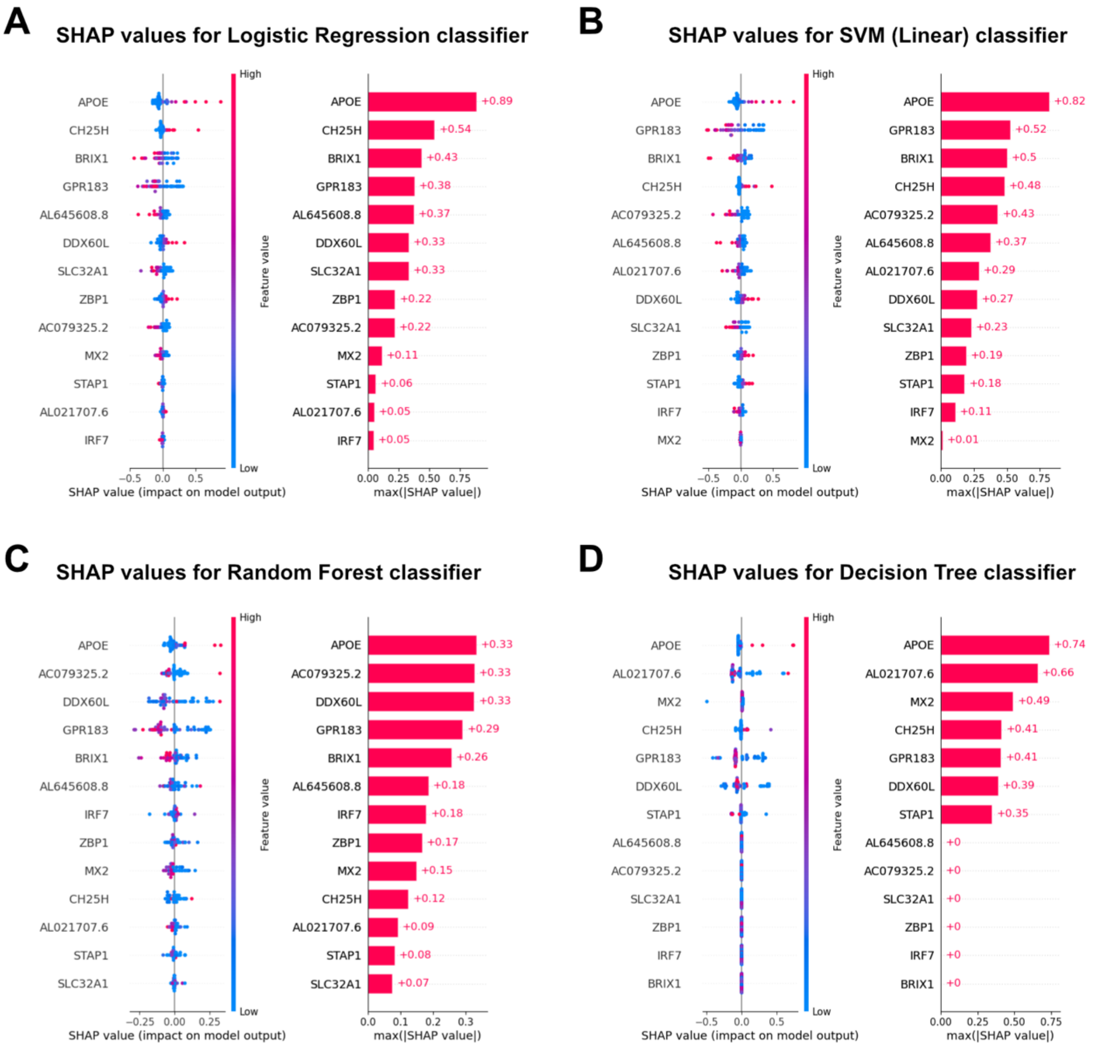

© 2024 Seitzman GD et al. *JAMA Ophthalmology*.

eTable 1. Characteristics of samples included in Phases I and II. 1 = corneal involvement; 0 = no corneal involvement

Sample	Site	DESEq	Machline Learning	RT-qPCR Validation	Corneal Involvement	Sex	Age
**sample_1**	Aravind, India	Yes	Yes	Yes	1	F	30
**sample_2**	Aravind, India	No	Yes	Yes	1	M	21
**sample_3**	Aravind, India	Yes	Yes	Yes	1	M	65
**sample_4**	Aravind, India	No	Yes	Yes	0	M	56
**sample_5**	Aravind, India	Yes	Yes	Yes	0	M	16
**sample_6**	Aravind, India	No	Yes	Yes	1	M	64
**sample_7**	Aravind, India	Yes	Yes	Yes	0	F	27
**sample_8**	Aravind, India	No	Yes	Yes	1	F	21
**sample_9**	Aravind, India	Yes	Yes	Yes	0	F	44
**sample_10**	Aravind, India	Yes	Yes	Yes	0	M	37
**sample_11**	Aravind, India	Yes	Yes	Yes	0	M	32
**sample_12**	Aravind, India	Yes	Yes	Yes	1	F	59
**sample_13**	Aravind, India	Yes	Yes	Yes	0	F	22
**sample_14**	Aravind, India	Yes	Yes	Yes	0	F	30
**sample_15**	Aravind, India	Yes	Yes	Yes	0	F	31
**sample_16**	Aravind, India	Yes	Yes	Yes	0	F	37
**sample_17**	Aravind, India	Yes	Yes	Yes	0	M	22
**sample_18**	Aravind, India	No	Yes	Yes	0	F	40
**sample_19**	Aravind, India	Yes	Yes	Yes	1	M	16
**sample_20**	Aravind, India	No	Yes	Yes	0	F	40
**sample_21**	Aravind, India	Yes	Yes	Yes	0	M	34
**sample_22**	Aravind, India	Yes	Yes	Yes	0	M	21
**sample_23**	Aravind, India	Yes	Yes	Yes	1	M	45
**sample_24**	Aravind, India	Yes	Yes	Yes	1	M	23
**sample_25**	Aravind, India	No	Yes	Yes	0	F	40
**sample_26**	Aravind, India	Yes	Yes	Yes	0	F	40
**sample_27**	Aravind, India	Yes	Yes	Yes	0	M	26
**sample_28**	Aravind, India	No	Yes	Yes	0	M	31
**sample_29**	Aravind, India	Yes	Yes	Yes	1	M	38
**sample_30**	Aravind, India	Yes	Yes	Yes	0	F	48
**sample_31**	Aravind, India	Yes	Yes	Yes	0	M	41
**sample_32**	Aravind, India	Yes	Yes	Yes	0	F	70
**sample_33**	Aravind, India	Yes	Yes	Yes	1	M	34
**sample_34**	Aravind, India	Yes	Yes	Yes	0	F	27
**sample_35**	Aravind, India	Yes	Yes	Yes	0	F	34
**sample_36**	Aravind, India	Yes	Yes	Yes	0	F	50
**sample_37**	Aravind, India	No	Yes	Yes	1	M	70
**sample_38**	Aravind, India	No	Yes	Yes	0	M	47
**sample_39**	Aravind, India	Yes	Yes	Yes	0	F	40

© 2024 Seitzman GD et al. *JAMA Ophthalmology*.

**sample_40**	Aravind, India	No	Yes	Yes	1	M	52
**sample_41**	Aravind, India	Yes	Yes	Yes	0	M	38
**sample_42**	Aravind, India	No	Yes	Yes	0	M	25
**sample_43**	Aravind, India	Yes	Yes	Yes	0	M	26
**sample_44**	Aravind, India	Yes	Yes	Yes	0	M	31
**sample_45**	Aravind, India	Yes	Yes	Yes	1	F	41
**sample_46**	Aravind, India	Yes	Yes	Yes	0	M	40
**sample_47**	Aravind, India	Yes	Yes	Yes	0	M	42
**sample_48**	Aravind, India	Yes	Yes	Yes	0	M	22
**sample_49**	UCSF, United States	No	Yes	No	1	F	30
**sample_50**	UCSF, United States	No	Yes	No	1	F	30
**sample_51**	UCSF, United States	No	Yes	No	0	F	38
**sample_52**	UCSF, United States	No	Yes	No	1	F	38
**sample_53**	UCSF, United States	No	Yes	No	1	F	13
**sample_54**	UCSF, United States	No	Yes	No	0	M	47
**sample_55**	UCSF, United States	No	Yes	No	0	M	47
**sample_56**	UCSF, United States	No	Yes	No	0	M	29
**sample_57**	UCSF, United States	No	Yes	No	1	F	83
**sample_58**	UCSF, United States	No	Yes	No	1	F	83

© 2024 Seitzman GD et al. *JAMA Ophthalmology*.

eTable 2. Differential gene expression between patients with and without corneal involvement

Gene Name	Gene ID	Log2FC	P-value	Adj. P-value
APOE	ENSG00000130203	2.17	<.001	.045
GPR183	ENSG00000169508	-1.68	<.001	.045
DDX60L	ENSG00000181381	-1.50	<.001	.048
CH25H	ENSG00000138135	-5.25	<.001	.048
BRIX1	ENSG00000113460	-1.81	<.001	.045
AL021707.6	ENSG00000272669	-2.76	<.001	.048
IRF7	ENSG00000185507	-1.63	<.001	.048
ZBP1	ENSG00000124256	-1.62	<.001	.048
SLC32A1	ENSG00000101438	-5.39	<.001	.048
STAP1	ENSG00000035720	-3.48	<.001	.048
AC079325.2	ENSG00000284690	-5.32	<.001	.048
MX2	ENSG00000183486	-1.57	<.001	.048
AL645608.8	ENSG00000272512	-2.97	<.001	.048

© 2024 Seitzman GD et al. *JAMA Ophthalmology.*

eTable 3. Characteristics of samples included in Phase III. 1 = corneal involvement; 0 = no corneal involvement

Sample	Site	Corneal involvement	Sex	Age
**sample_59**	Aravind, India	0	F	31
**sample_60**	Aravind, India	0	F	31
**sample_61**	Aravind, India	0	F	56
**sample_62**	Aravind, India	0	M	33
**sample_63**	Aravind, India	0	M	33
**sample_64**	Aravind, India	0	M	unknown
**sample_65**	Aravind, India	1	F	42
**sample_66**	Aravind, India	1	F	42
**sample_67**	Aravind, India	0	F	49
**sample_68**	Aravind, India	0	M	61
**sample_69**	Aravind, India	0	M	47
**sample_70**	Aravind, India	0	M	45
**sample_71**	Aravind, India	0	F	55
**sample_72**	Aravind, India	0	F	55
**sample_73**	Aravind, India	0	M	19
**sample_74**	Aravind, India	0	M	19
**sample_75**	Aravind, India	0	M	25
**sample_76**	Aravind, India	0	M	56
**sample_77**	Aravind, India	0	M	35
**sample_78**	Aravind, India	1	M	33
**sample_79**	Aravind, India	0	M	21
**sample_80**	Aravind, India	0	M	63
**sample_81**	Aravind, India	0	M	63
**sample_82**	Aravind, India	1	M	53
**sample_83**	Aravind, India	0	M	33
**sample_84**	Aravind, India	1	F	41
**sample_85**	Aravind, India	0	F	27
**sample_86**	Aravind, India	1	M	35
**sample_87**	Aravind, India	0	M	35
**sample_88**	Aravind, India	0	F	35
**sample_89**	Aravind, India	1	F	35
**sample_90**	Aravind, India	0	M	62
**sample_91**	Aravind, India	0	F	68
**sample_92**	Aravind, India	0	M	44

© 2024 Seitzman GD et al. *JAMA Ophthalmology*.

**sample_93**	Aravind, India	0	M	28
**sample_94**	Aravind, India	0	M	50
**sample_95**	Aravind, India	0	M	50
**sample_96**	Aravind, India	1	M	60
**sample_97**	Aravind, India	0	M	32
**sample_98**	Aravind, India	0	M	55
**sample_99**	Aravind, India	0	M	22
**sample_100**	Aravind, India	0	M	22
**sample_101**	Aravind, India	0	M	59
**sample_102**	Aravind, India	0	M	42
**sample_103**	Aravind, India	0	M	38
**sample_104**	Aravind, India	0	M	35
**sample_105**	Aravind, India	0	M	46
**sample_106**	Aravind, India	0	M	24
**sample_107**	Aravind, India	0	M	52
**sample_108**	Aravind, India	0	M	32
**sample_109**	Aravind, India	0	M	40
**sample_110**	Aravind, India	0	M	40
**sample_111**	Aravind, India	0	F	31
**sample_112**	Aravind, India	0	M	32
**sample_113**	Aravind, India	0	F	51
**sample_114**	Aravind, India	0	M	22
**sample_115**	Aravind, India	0	F	35
**sample_116**	Aravind, India	0	F	59
**sample_117**	Aravind, India	0	M	31
**sample_118**	Aravind, India	0	F	34
**sample_119**	Aravind, India	0	M	39
**sample_120**	Aravind, India	1	M	35
**sample_121**	Aravind, India	1	M	35
**sample_122**	Aravind, India	0	F	27
**sample_123**	Aravind, India	0	F	65
**sample_124**	Aravind, India	1	F	37
**sample_125**	Aravind, India	0	F	37
**sample_126**	Aravind, India	1	F	34
**sample_127**	Aravind, India	0	F	34
**sample_128**	Aravind, India	0	M	73
**sample_129**	Aravind, India	0	M	73
**sample_130**	Aravind, India	0	F	21
**sample_131**	Aravind, India	0	M	21

© 2024 Seitzman GD et al. *JAMA Ophthalmology*.

**sample_132**	Aravind, India	0	M	21
**sample_133**	Aravind, India	0	M	32
**sample_134**	Aravind, India	0	M	38
**sample_135**	Aravind, India	0	M	unknown
**sample_136**	Aravind, India	0	M	62
**sample_137**	Aravind, India	0	F	34
**sample_138**	Aravind, India	0	M	61
**sample_139**	Aravind, India	0	M	26
**sample_140**	Aravind, India	0	F	38
**sample_141**	Aravind, India	0	F	38
**sample_142**	Aravind, India	0	F	19
**sample_143**	Aravind, India	0	F	19
**sample_144**	Aravind, India	0	M	22
**sample_145**	Aravind, India	1	M	71
**sample_146**	Aravind, India	0	M	71
**sample_147**	Aravind, India	0	F	21
**sample_148**	Aravind, India	0	F	21
**sample_149**	Aravind, India	0	F	71
**sample_150**	Aravind, India	0	M	40
**sample_151**	Aravind, India	1	F	11
**sample_152**	Aravind, India	1	F	11
**sample_153**	Aravind, India	0	F	30
**sample_154**	Aravind, India	0	M	33
**sample_155**	Aravind, India	0	M	33
**sample_156**	Aravind, India	0	M	48
**sample_157**	Aravind, India	1	M	48
**sample_158**	Aravind, India	0	M	32
**sample_159**	Aravind, India	0	M	32
**sample_160**	Aravind, India	0	F	42
**sample_161**	Aravind, India	0	M	29
**sample_162**	Aravind, India	0	M	29
**sample_163**	Aravind, India	0	F	30
**sample_164**	Aravind, India	0	M	22
**sample_165**	Khon Kaen University, Thailand	1	F	66
**sample_166**	Khon Kaen University, Thailand	0	F	47
**sample_167**	Khon Kaen University, Thailand	0	F	64
**sample_168**	Khon Kaen University, Thailand	1	M	22
**sample_169**	Khon Kaen University, Thailand	0	F	80

© 2024 Seitzman GD et al. *JAMA Ophthalmology*.

**sample_170**	Khon Kaen University, Thailand	0	M	37
**sample_171**	Khon Kaen University, Thailand	0	M	89
**sample_172**	Khon Kaen University, Thailand	0	M	69
**sample_173**	Khon Kaen University, Thailand	0	F	57
**sample_174**	Khon Kaen University, Thailand	0	F	30
**sample_175**	Phramongkutklao Hospital, Thailand	0	M	35
**sample_176**	Phramongkutklao Hospital, Thailand	1	M	21
**sample_177**	Phramongkutklao Hospital, Thailand	1	M	21
**sample_178**	Phramongkutklao Hospital, Thailand	1	M	30
**sample_179**	Phramongkutklao Hospital, Thailand	1	M	22
**sample_180**	Phramongkutklao Hospital, Thailand	1	F	27
**sample_181**	Phramongkutklao Hospital, Thailand	1	M	28
**sample_182**	Phramongkutklao Hospital, Thailand	1	M	20
**sample_183**	Phramongkutklao Hospital, Thailand	1	M	22
**sample_184**	Phramongkutklao Hospital, Thailand	0	M	25
**sample_185**	Phramongkutklao Hospital, Thailand	0	F	32
**sample_186**	Phramongkutklao Hospital, Thailand	1	F	44
**sample_187**	Phramongkutklao Hospital, Thailand	1	M	17
**sample_188**	Phramongkutklao Hospital, Thailand	1	M	80
**sample_189**	Phramongkutklao Hospital, Thailand	1	M	16
**sample_190**	Phramongkutklao Hospital, Thailand	1	M	39
**sample_191**	Phramongkutklao Hospital, Thailand	0	M	25
**sample_192**	Phramongkutklao Hospital, Thailand	1	M	21
**sample_193**	Phramongkutklao Hospital, Thailand	1	M	22
**sample_194**	Phramongkutklao Hospital, Thailand	1	M	21
**sample_195**	Phramongkutklao Hospital, Thailand	1	M	50
**sample_196**	Chulalongkorn University, Thailand	0	F	75
**sample_197**	Chulalongkorn University, Thailand	0	F	27
**sample_198**	Chulalongkorn University, Thailand	1	M	20
**sample_199**	Chulalongkorn University, Thailand	0	F	25
**sample_200**	Chulalongkorn University, Thailand	0	M	23
**sample_201**	Chulalongkorn University, Thailand	1	M	33
**sample_202**	Chulalongkorn University, Thailand	0	M	59
**sample_203**	Chulalongkorn University, Thailand	0	F	49

© 2024 Seitzman GD et al. *JAMA Ophthalmology*.

**sample_204**	Chulalongkorn University, Thailand	0	M	53
**sample_205**	Chulalongkorn University, Thailand	1	M	20
**sample_206**	Chulalongkorn University, Thailand	0	M	32
**sample_207**	Chulalongkorn University, Thailand	1	F	42
**sample_208**	Chulalongkorn University, Thailand	0	M	25
**sample_209**	Chulalongkorn University, Thailand	1	M	32
**sample_210**	Chulalongkorn University, Thailand	0	M	28
**sample_211**	Chulalongkorn University, Thailand	1	M	51
**sample_212**	Chulalongkorn University, Thailand	1	M	23
**sample_213**	Chulalongkorn University, Thailand	0	F	59
**sample_214**	Chulalongkorn University, Thailand	1	F	52
**sample_215**	Chulalongkorn University, Thailand	1	M	31
**sample_216**	Chulalongkorn University, Thailand	0	F	38
**sample_217**	Chulalongkorn University, Thailand	0	F	48
**sample_218**	Chulalongkorn University, Thailand	1	F	18
**sample_219**	Chulalongkorn University, Thailand	0	M	63
**sample_220**	Chulalongkorn University, Thailand	1	F	39
**sample_221**	Chulalongkorn University, Thailand	0	F	62
**sample_222**	Chulalongkorn University, Thailand	0	F	29
**sample_223**	Chulalongkorn University, Thailand	0	M	29

© 2024 Seitzman GD et al. *JAMA Ophthalmology*.
