# Supplementary material for: Biomarker Detection and Validation for Corneal Involvement in Patients With Acute Infectious Conjunctivitis
Source: JAMA Ophthalmol. 2024 Aug 15;142(9):865–71. doi: 10.1001/jamaophthalmol.2024.2891 (PMC11327903; doi:10.1001/jamaophthalmol.2024.2891)
Supplement: Supplement 2. — The SCORPIO Study Group members [file jamaophthalmol-e242891-s002.pdf]

Supplemental Online Content: Nonauthor Collaborators
*First name, last name, and suffix (if applicable) are required and will appear in PubMed.

*Group Name(s): SCORPIO Study Group

*First Name and Middle Initial(s)	*Last Name	*Suffix (eg, Jr, III)	Academic Degrees	Institution	Location (city, state/province, country)	Role or Contribution, eg, chair, principal investigator	Group (if more than 1 Group listed in the byline) and/or Subgroup (eg, Steering Committee)
Ramesh	Gunasekaran		MS	Aravind Eye Hospital	Madurai, Tamil Nadu, India	Study Coordinator	
Sankalp Singh	Sharma		DO, DNB, RFCO	Aravind Eye Hospital	Madurai, Tamil Nadu, India	Co-Investigator	
Vishnu	Teja		MD	Aravind Eye Hospital	Madurai, Tamil Nadu, India	Co-Investigator	
Meenu	Chaudhary		MD	B.P Koirala Lions Center for Ophthalmic Studies	Kathmandu, Nepal	Co-Investigator	
Sanjeeta	Situala		MD, MBBS	B.P Koirala Lions Center for Ophthalmic Studies	Kathmandu, Nepal	Co-Investigator	
Ali	Sié		MD, PhD	Centre de Recherche en Sante de Nouna	Nouna, Burkina Faso	Co-Investigator	
Boubacar	Coulibaly		MD, PhD	Centre de Recherche en Sante de Nouna	Nouna, Burkina Faso	Co-Investigator	
Mamadou	Bountogo		MD	Centre de Recherche en Sante de Nouna	Nouna, Burkina Faso	Co-Investigator	
Thanapong	Somkijrungroj		MD	Chulalongkorn University	Bangkok, Thailand	Co-Investigator	
Huy	Tran		MD, MSc	Hai Yen Vision Institute	Ho Chi Minh City, Vietnam	Co-Investigator	
Linh Hoàng	Mai		MD, MSc	Hai Yen Vision Institute	Ho Chi Minh City, Vietnam	Co-Investigator	
Thảo Hạ	Xuân		MD	Hai Yen Vision Institute	Ho Chi Minh City, Vietnam	Co-Investigator	
Yen	Tran		MD, PhD	Hai Yen Vision Institute	Ho Chi Minh City, Vietnam	Co-Investigator	
Cristhian A.	Urzua		MD, MSc	Hospital Clinico Universidad de Chile	Santiago, Chile	Co-Investigator	
Fabian	Vega		MD	Hospital Clinico Universidad de Chile	Santiago, Chile	Co-Investigator	
Felipe	Salgado		MD	Hospital Clinico Universidad de Chile	Santiago, Chile	Co-Investigator	
Loreto	Cuitino		MD, PhD	Hospital Clinico Universidad de Chile	Santiago, Chile	Co-Investigator	
Fernando Pérez	Pérez		MD	Instituto Mexicano de Oftalmología	Santiago de Queretaro, Queretaro, Mexico	Co-Investigator	
Jaime Macías	Martínez		MD	Instituto Mexicano de Oftalmología	Santiago de Queretaro, Queretaro, Mexico	Co-Investigator	
Van Charles	Lansingh		MD, PhD	Instituto Mexicano de Oftalmología	Santiago de Queretaro, Queretaro, Mexico	Co-Investigator	
Sukhumal	Thanapaisal		MD	Khon Kaen University	Khon Kaen, Thailand	Co-Investigator	
George	McKie		DVM, PhD	National Eye Institute		Program Officer	
De-Kuang	Hwang		MD, PhD	National Yang Min Chiao Tung University School of Medicine	Taipei, Taiwan	Co-Investigator	
Nai-Wen	Fan		MD	National Yang Min Chiao Tung University School of Medicine	Taipei, Taiwan	Co-Investigator	
Kenia	Chavez		BS	Oregon Health and Science University	Portland, Oregon, USA	Study Coordinator	
Travis	Redd		MD, MPH	Oregon Health and Science University	Portland, Oregon, USA	Co-Investigator	
Winston	Chamberlain		MD	Oregon Health and Science University	Portland, Oregon, USA	Co-Investigator	
Angel	Cheng		BS	Pacific Vision Institute of Hawaii	Honolulu, Hawaii, USA	Study Coordinator	
Vivien	Tham		MD	Pacific Vision Institute of Hawaii	Honolulu, Hawaii, USA	Co-Investigator	
Alexandra	Bograd		MD	Pallas Kliniken, Department of Ophthalmology	Olten, Switzerland	Co-Investigator	
Christoph	Tappeiner		MD	Pallas Kliniken, Department of Ophthalmology	Olten, Switzerland	Co-Investigator	
David	Goldblum		MD	Pallas Kliniken, Department of Ophthalmology	Olten, Switzerland	Co-Investigator	
Abba Kaka Hajia	Yakoura		MD	Programme National de Santé Oculaire	Niamey, Niger	Co-Investigator	
Abdou	Amza		MD	Programme National de Santé Oculaire	Niamey, Niger	Co-Investigator	
Abdoul Salam	Youssoufou Souley		MD	Programme National de Santé Oculaire	Niamey, Niger	Co-Investigator	
Adam	Nouhou Diori		MD	Programme National de Santé Oculaire	Niamey, Niger	Co-Investigator	
Boubacar	Kadri		MD	Programme National de Santé Oculaire	Niamey, Niger	Co-Investigator	
Boubacar	Mariama		MD	Programme National de Santé Oculaire	Niamey, Niger	Co-Investigator	
Cissé Mamadou	Ibrahim		MD	Programme National de Santé Oculaire	Niamey, Niger	Co-Investigator	
Lamine Aboubacar	Roufaye		MD	Programme National de Santé Oculaire	Niamey, Niger	Co-Investigator	

Supplemental Online Content: Nonauthor Collaborators
*First name, last name, and suffix (if applicable) are required and will appear in PubMed.

***First Name and Middle Initial(s)**	***Last Name**	***Suffix (eg, Jr, III)**	Academic Degrees	Institution	Location (city, state/province, country)	Role or Contribution, eg, chair, principal investigator	Group (if more than 1 Group listed in the byline) and/or Subgroup (eg, Steering Committee)
Nassirou	Beido		MS	Programme National de Santé Oculaire	Niamey, Niger	Study Coordinator	
Ramatou	Boulhassane		MD	Programme National de Santé Oculaire	Niamey, Niger	Co-Investigator	
Saley	Ali		MD	Programme National de Santé Oculaire	Niamey, Niger	Co-Investigator	
Zakou	Abdou		MD	Programme National de Santé Oculaire	Niamey, Niger	Co-Investigator	
Etian	Livny		MD	Rabin Medical Center	Petah Tikva, Israel	Co-Investigator	
Irit	Bahar		MD, MHA	Rabin Medical Center	Petah Tikva, Israel	Co-Investigator	
Lee	Goren		MD	Rabin Medical Center	Petah Tikva, Israel	Co-Investigator	
Ruti	Sella		MD	Rabin Medical Center	Petah Tikva, Israel	Co-Investigator	
Clare	Kelliher		MD	Sinai Hospital	Baltimore, Maryland, USA	Co-Investigator	
Laura	Green		MD	Sinai Hospital	Baltimore, Maryland, USA	Co-Investigator	
Hong Shing	Ong		MBBS, FRCOpth	Singapore Eye Research Institute	Singapore, Singapore	Co-Investigator	
Jodhbir S.	Mehta		MBBS, PhD	Singapore Eye Research Institute	Singapore, Singapore	Co-Investigator	
Yu-Chi	Liu		MD, MCI, PhD	Singapore Eye Research Institute	Singapore, Singapore	Co-Investigator	
Hong Sheng	Chiong		MB BCh BAO, FRANZCO	The University of Sydney, Save Sight Institute	Sydney, New South Wales, Australia	Co-Investigator	
Javier	Lacorzana		MD, PhD, FEBO	The University of Sydney, Save Sight Institute	Sydney, New South Wales, Australia	Co-Investigator	
Maria	Cabrera-Aguas		MBBS, MIPH, PhD	The University of Sydney, Save Sight Institute	Sydney, New South Wales, Australia	Co-Investigator	
Stephanie	Watson		BSc, MBBS, PhD, FRANZCO	The University of Sydney, Save Sight Institute	Sydney, New South Wales, Australia	Co-Investigator	
Kuniyoshi	Kanai		OD, FAAO	University of California Berkeley School of Optometry	Berkeley, California, USA	Co-Investigator	
Meredith	Whiteside		OD, FAAO	University of California Berkeley School of Optometry	Berkeley, California, USA	Co-Investigator	
Edmund	Tsui		MD, MS	University of California Los Angeles Stein Eye Institute	Los Angeles, California, USA	Co-Investigator	
Nina M.	Cherian		BS	University of California Los Angeles Stein Eye Institute	Los Angeles, California, USA	Study Coordinator	
Rachel	Feit-Leichman		MD	University of California Los Angeles Stein Eye Institute	Los Angeles, California, USA	Co-Investigator	
Reginald E.	Hughes	Jr.	MD	University of California Los Angeles Stein Eye Institute	Los Angeles, California, USA	Co-Investigator	
Tania	Onclinx		MD	University of California Los Angeles Stein Eye Institute	Los Angeles, California, USA	Co-Investigator	
Jose Torres	Salgado		BS	University of California Los Angeles Stein Eye Institute	Los Angeles, California, USA	Study Coordinator	
Joseph K.	Privratsky		BS	University of California Los Angeles Stein Eye Institute	Los Angeles, California, USA	Study Coordinator	
Carol	Yu		OD	University of California San Diego Shiley Eye Institute	La Jolla, California, USA	Co-Investigator	
Esmeralda	McClean		OD	University of California San Diego Shiley Eye Institute	La Jolla, California, USA	Co-Investigator	
Iliana	Molina		OD	University of California San Diego Shiley Eye Institute	La Jolla, California, USA	Co-Investigator	
Philip	Kim		OD	University of California San Diego Shiley Eye Institute	La Jolla, California, USA	Co-Investigator	
Danny	Yu		BS	University of California San Francisco Francis I. Proctor Foundatior	San Francisco, California, USA		
Elodie	Lebas		RN	University of California San Francisco Francis I. Proctor Foundatior	San Francisco, California, USA		
Emily	Colby		MPH, BS	University of California San Francisco Francis I. Proctor Foundatior	San Francisco, California, USA		
Thomas	Abraham		BS	University of California San Francisco Francis I. Proctor Foundatior	San Francisco, California, USA		
YuHeng	Liu		BS	University of California San Francisco Francis I. Proctor Foundatior	San Francisco, California, USA		
Steven	Yeh		MD, FASRS	University of Nebraska Medical Center Truhlsen Eye Institute	Omaha, Nebraska, USA	Co-Investigator	
Tolulope	Fashina		MD	University of Nebraska Medical Center Truhlsen Eye Institute	Omaha, Nebraska, USA	Study Coordinator	
James	Chodosh		MD, MPH	University of New Mexico	Albuquerque, New Mexico, USA		
Bridgit	Tarkap			University of Papua New Guinea School of Medicine and Health Sc	Port Moresby, Papua New Guinea	Study Coordinator	
Jambi N.	Garap		MBBS, MMED (Ophthal)	University of Papua New Guinea School of Medicine and Health Sc	Port Moresby, Papua New Guinea	Co-Investigator	
Magdalene	Mangot		MBBS	University of Papua New Guinea School of Medicine and Health Sc	Port Moresby, Papua New Guinea	Co-Investigator	

Supplemental Online Content: Nonauthor Collaborators
*First name, last name, and suffix (if applicable) are required and will appear in PubMed.

***First Name and Middle Initial(s)**	***Last Name**	***Suffix (eg, Jr, III)**	Academic Degrees	Institution	Location (city, state/province, country)	Role or Contribution, eg, chair, principal investigator	Group (if more than 1 Group listed in the byline) and/or Subgroup (eg, Steering Committee)
Edwin	Amel			Vanuatu Eye Program, Ministry of Health	Luganville Santo, Vanuatu	Co-Investigator	
Fasihah	Taleo		DAP&E, BS	Vanuatu Eye Program, Ministry of Health	Luganville Santo, Vanuatu	Study Coordinator	
Johnson	Kasso		MD	Vanuatu Eye Program, Ministry of Health	Luganville Santo, Vanuatu	Co-Investigator	
Kalbule	Willie		MD	Vanuatu Eye Program, Ministry of Health	Luganville Santo, Vanuatu	Co-Investigator	
Madopule	Nanu			Vanuatu Eye Program, Ministry of Health	Luganville Santo, Vanuatu	Co-Investigator	
Prudence	Rymill			Vanuatu Eye Program, Ministry of Health	Luganville Santo, Vanuatu	Study Coordinator	
Anthony W.	Solomon		MBBS, DTM&H, PhD, PGCAP, FHEA, FRCP	World Health Organization			
