# Supplementary material for: Biomarker Detection and Validation for Corneal Involvement in Patients With Acute Infectious Conjunctivitis
Source: JAMA Ophthalmol. 2024 Aug 15;142(9):865–71. doi: 10.1001/jamaophthalmol.2024.2891 (PMC11327903; doi:10.1001/jamaophthalmol.2024.2891)
Supplement: Supplement 3. — Data sharing statement [file jamaophthalmol-e242891-s003.pdf]

Data Sharing Statement

Seitzman. Biomarker Detection and Validation for Corneal Involvement in Patients With Acute Infectious Conjunctivitis. *JAMA Ophthalmol*. Published August 15, 2024.
doi:10.1001/jamaophthalmol.2024.2891

Data
Data available: Yes
Data types: Other (please specify)
Additional Information: Relevant de-identified data can be accessed https://datadryad.org/stash at publication.
How to access data: https://datadryad.org/stash
When available: With publication

Supporting Documents
Document types: None

Additional Information
Who can access the data: Researchers whose proposed use of the data has been approved
Types of analyses: For a specified purpose
Mechanisms of data availability: With a signed data access agreement
